# Associations between adherence to MIND diet and general obesity and lipid profile: A cross-sectional study

**DOI:** 10.3389/fnut.2023.1078961

**Published:** 2023-04-11

**Authors:** Hawal Lateef Fateh, Sarmad S. Muhammad, Negin Kamari

**Affiliations:** ^1^Nursing Department, Kalar Technical College, Sulaimani Polytechnic University, Sulaymaniyah, Iraq; ^2^Medical Laboratory Technology Department, Kalar Technical College, Sulaimani Polytechnic University, Sulaymaniyah, Iraq; ^3^Department of Nutritional Sciences, School of Nutritional Sciences and Food Technology, Kermanshah University of Medical Sciences, Kermanshah, Iran

**Keywords:** MIND diet, lipid profile, general obesity, Kurdish adult, cross-sectional

## Abstract

**Background:**

The term “Mediterranean-DASH Intervention for Neurodegenerative Delay (MIND)” has recently been coined to describe a new eating pattern. Recent research is looking at how this food pattern affects chronic illnesses. Thus, this study aimed to investigate the association between the use and adherence to the MIND diet with general obesity and blood lipid profile.

**Methods:**

In this cross-sectional study, 1,328 Kurdish adults between the ages of 39 and 53 had their dietary intake evaluated using a valid and reliable 168-item Food Frequency Questionnaire (FFQ). Adherence to the MIND diet was examined based on the components advised in this eating pattern. Each subject’s lipid profiles and anthropometric measurements were documented.

**Results:**

The mean age and BMI in the study population were 46.16 ± 7.87 year and 27.19 ± 4.60 kg/m^2^, respectively. The chances of having increased serum triglycerides (TG) were 42% lower in those in the third tertile of the MIND diet score compared to those in the first tertile (ORs: 0.58; 95% CI 0.38−0.95; *P* = 0.001). In the crude model and after adjusting for confounders, lowering high-density lipoprotein cholesterol (HDL-C) (ORs: 0.72; 95% CI 0.55−1.15; *P* = 0.001).

**Conclusion:**

We found that greater adherence to the MIND diet was associated with the decrease odds of general obesity and lipid profile. Further study is essential owing to the relevance of chronic diseases like MetS and obesity in health status.

## Introduction

Worldwide, the incidence of obesity is significantly rising and is a serious public health problem. It is described as an accumulation of extra body fat that causes serious medical conditions (1, 2). The prevalence of overweight and obesity is high, both in developed and developing countries. In several local surveys in subregions and clinical populations in Iraq, high proportions of obesity have been reported. In a community-based survey (*N* = 1,480 adults in 2017) in Erbil city, Iraq, the prevalence of overweight was 33.4% and obesity was 40.9%, and in Basrah, Southern Iraq (2003–2010), overweight/obesity was 55.1% (3).

Diet is a modifiable risk factor for obesity. Several research have studied the links between adherence to healthy dietary patterns including Dietary Approaches to Stop Hypertension (DASH) diet (4) and the Mediterranean diet (MD) (5). According to specific research, following the DASH diet was strongly related to body mass index (BMI), serum triglycerides (TG), and HDL cholesterol (6). While adherence to the MD diet was inversely linked with obesity and BMI in the Spanish population, it was not associated with BMI in a large sample of Europeans (7). Moreover, central obesity was negatively correlated with DASH diet adherence, but not overall obesity (8). After 2 months of management with the DASH diet, patients with non-alcoholic fatty livers reported lower weight and BMI (9).

The MIND (Mediterranean-DASH Intervention for Neurodegenerative Delay) diet, based on nourishing and harmful foods for the brain, has recently been proposed (10). It includes 15 components, of which 10 are brain-healthy foods (green leafy vegetables, other vegetables, berries, nuts, beans, whole grains, fish, poultry, olive oil, and wine) and 5 are brain-unhealthy foods (cheese, butter or margarine, fast foods or fried foods, red meat, and pastries or sweets). This food pattern has yet to be extensively studied in the context of chronic diseases. Most studies have discovered a link between strong adherence to this diet and physical activity (PA) and slower cognitive aging, reduced risk of Alzheimer’s disease (11–14). Although the relationship between the DASH and MD diets and obesity has been separately assessed (15, 16), no study is available linking the MIND diet to obesity and lipid profiles. Therefore, we aimed to investigate the association between the use and adherence to the MIND diet with general obesity and blood lipid profiles in Kurdish adults.

## Materials and methods

### Study design and participants

This cross-sectional study was performed on 1,328 Kurdish adults, who referred to Health centers in Kalar, Sulaymaniyah, and Erbil city. After the random selection of 3 health centers from 18 health centers in these three cities, an identical number of subjects were randomly chosen from each center. We included only healthy participants in the current study since CVD, diabetes, thyroid diseases, pregnancy, etc., significantly affect general obesity and lipid profile. In addition, these conditions seem to lead to changes in diet and specific medication use.

Moreover, due to the possibility of under- or over-reporting of dietary intake, individuals with a total energy intake outside the range of 800–4,200 kcal/day for men and 600–3,500 kcal/day for women were excluded. Also, subjects with missing data were excluded from the study.

The study staff (including a medical doctor, laboratory technicians, executive manager, and receptionist) obtained the primary data, including demographics (education level, age, sex, residency, marital status, socio economic status (SES), smoking, alcohol use, dietary intake, physical activity (PA) [very low: <600, low: 600–3000, and moderate and high >3,000 MET-min/week] (11), anthropometric indices, and body composition).

### Anthropometry

A bioimpedance analyzer (Inbody 770, Inbody Co, Seoul, Korea) was used to determine body weight to the nearest 0.1 kg. BSM 370 (Biospace Co, Seoul, Korea) was used to measure height. The formula for determining a person’s body mass index (BMI) is to multiply their weight in kilograms by the square of their height (in meters).

### Blood sampling and biochemical assays

7 mL of blood samples were taken and placed into the clot tubes after 8–12 h of fasting. Serum samples were centrifuged at 4°C for 10–15 min, then kept at −80°C until bioanalysis. TG, HDL-C, LDL-C and total cholesterol, and serum concentrations were assessed using commercially available assays according to the manufacturer’s instructions.

### Dietary assessment

Usual dietary intake was estimated using a valid and reliable 168-item Food Frequency Questionnaire (FFQ) (17) which included a list of groceries and a standard size of each food item and was administered by skilled dietitians *via* face-to-face interviews.

Participants stated how often they consumed each substance daily, weekly, monthly, and yearly.

Household measurements were used to convert the amount of food eaten to grams (18) and calculated using a modified version of NUTRITIONIST IV software for Kurdish foods (version 7.0; N-Squared Computing, Salem, OR, USA).

### Construction of the MIND diet score

Data derived from the FFQ was utilized for the development of the MIND diet score. Components of the MIND diet that we used in this study are presented in Table 1. In the original scoring method of the MIND diet (10), 15 dietary parameters were considered. Ten brain-healthy food categories (green leafy vegetables, other vegetables, nuts, berries, beans, whole grains, fish, chicken, olive oil, and wine) and five brain-unhealthy food groups (red meats, butter and stick margarine, cheese, pastries and sweets, and fast/fried food) were studied. Because of a lack of information in the original data set, olive oil intake was not included in the current study, and wine was eliminated since Iraqi inhabitants are Muslims, and the use of wine is prohibited. Participants were categorized into tertile categories of consumption of these components to create the MIND diet score. Individuals in the third tertiles who consumed the most brain-healthy food groups, such as green leafy vegetables, other vegetables, nuts, berries, beans, whole grains, fish, and poultry, received a score of 1, those in the middle tertile received a score of 0.5, and those in the lowest tertile received a score of 0. Regarding brain-unhealthy food groups including red meats, butter and stick margarine, cheese, pastries and sweets, and fast/fried food intake, individuals in the lowest tertile were given the score of 1, those in the middle tertile were assigned the score of 0.5 and participants with the highest consumption of these food groups were given the score of 0. Ultimately, the MIND diet’s total score was obtained by adding the scores of its components. As a result, the total MIND diet score varied between 0 and 12.5.

**TABLE 1:** Mediterranean-DASH Intervention for Neurodegenerative Delay (MIND) diet components.

Brain healthy foods	
Green leafy vegetables	Cabbage, greens, lettuce
Other vegetables	Green/red peppers, raw carrot, potato, peas or lima beans, tomatoes, tomato sauce, eggplant, onion, cucumber
Berries	Strawberries (strawberries, cherries, fresh berries)
Nuts	Walnuts, pistachios, hazelnuts, almonds, peanuts
Whole grains	Dark bread
Fish	Fish
Beans	Beans, lentils, peas, chick pea, mung bean
Poultry	Chicken
**Brain unhealthy foods**	
Butter, margarine	Butter, margarine, animal fats
Cheese	Cheese
Red meat and products	Red meat, hamburgers, sausages
Fast fried foods	French fries, pizza
Pastries and sweets	Biscuit, cake, chocolate, ice cream, confections, cocoa

### Statistical analysis

Version 14.2 of STATA software was used for the analysis (StataCorp, College Station, TX, USA). At 0.05, the significance level was established. Using a standard BMI cut-off for each sex, data were presented as mean, standard deviation, and percentages (frequency) for quantitative and qualitative characteristics, respectively. For continuous and categorical variables, one-way analysis of variance (ANOVA) and χ^2^ tests were used, respectively, to analyze the characteristics of the study participants by the MIND diet score tertiles. Logistic regression was used to assess the odds ratio and 95% confidence intervals for the link between the MIND diet score and obesity and lipid profile. Three adjusted models and a crude model each revealed the risk. This study’s first tertile of exposure was used as the reference category.

### Ethical approval

The Ethics Committee of Sulaimani Polytechnic University, Kalar technical college approved the study (No: KTC: 0629092022). Both verbal and written informed permission was obtained from each participant. All procedures followed the necessary rules and regulations. The Declaration of Helsinki authorized the conduct of this research.

## Results

Of the 1,328 participants in the study, 686 (51.60%) were female. Most of the participants 637 (47.96%) had moderate physical activity. Significant differences were observed between the three tertiles in terms of BMI, TG, and HDL-C, (*P* < 0.001). Participants in the third tertile had lower TG and BMI than participants in the second and first tertiles, (*P* < 0.001) (Table 2).

**TABLE 2:** General characteristics of the participants in the study based on tertiles (T) of MIND.

Variables	Total	Tertiles of MIND diet score		
		**T1** **(score<6)**	**T2** **(score 6−7.5)**	**T3** **(score 7.5−12.5)**
Subjects, *n*	1,328	531	422	375
Age (years)	46.16 ± 7.87	46.93 ± 7.99	45.85 ± 7.89	45.42 ± 7.58*
**Sex, *n* (%)**				
Male	642 (48.40)	243 (45.93)	209 (49.66)	190 (50.48)*
Female	686 (51.60)	288 (54.07)	213 (50.34)	185 (49.52)*
**Residency, *n* (%)**				
Urban	791 (59.63)	237 (44.73)	264 (62.73)	290 (77.25)*
Rural	537 (40.37)	294 (55.27)	158 (37.27)	85 (22.75)*
**Marital status, *n* (%)**				
Married	1192 (89.74)	471 (88.51)	382 (90.71)	339 (90.39)*
Single	70 (5.27)	33 (6.24)	21 (4.86)	16 (4.35)*
Divorced and other	66 (4.99)	27 (5.25)	19 (4.43)	20 (5.27)*
**Socio-economic status, *n* (%)**				
1 (lowest)	428 (32.29)	233 (43.95)	124 (29.41)	71 (19.00)*
2	437 (32.90)	175 (32.90)	149 (35.33)	113 (30.22)*
3 (highest)	463 (34.81)	123 (23.15)	149 (35.33)	191 (50.77)*
**Physical activity (met h/day)**				
Low	392 (29.53)	142 (26.70)	129 (30.70)	121 (32.22)*
Moderate	637 (47.96)	246 (46.44)	203 (47.94)	188 (50.14)*
High	299 (22.51)	143 (26.87)	90 (21.37)	66 (17.63)*
Current smoker, *n* (%)	157 (11.86)	74 (13.85)	46 (10.88)	37 (10.13)*
Alcohol use, *n* (%)	61 (4.58)	21 (3.95)	20 (4.73)	20 (5.31)
BMI (kg/m^2^)	27.19 ± 4.60	28.02 ± 4.43	27.13 ± 4.49	26.65 ± 4.71*
TG (mg/dl)	133.08 ± 79.60	142.23 ± 86.57	133.97 ± 79.78	125.94 ± 73.43*
T-C, (mg/dl)	184.89 ± 37.16	184.99 ± 35.66	185.21 ± 37.62	184.57 ± 36.61
LDL-C (mg/dl)	101.74 ± 25.02	101.70 ± 23.81	101.83 ± 25.17	101.71 ± 25.72
HDL-C (mg/dl)	46.63 ± 11.35	45.25 ± 10.98	46.68 ± 11.49	47.55 ± 11.40*

Data are shown mean ± SD for continuous variables and *n* (%) categorical variables.

*P*-value was obtained one-way ANOVA and Chi-square test.

BMI, body mass index; HDL-C, high-density lipoprotein cholesterol; LDL-C, low-density lipoprotein cholesterol; TG, triglycerides; T-C, total cholesterol.

*The association was statically significant.

Table 3 shows the daily intake of dietary types, macronutrients, and micronutrients according to the tertials of the MIND diet score. After adjusting for energy intake, the highest tertials of the MIND diet score had significantly higher daily intakes of whole grains, refined grains, legumes, dairy, fruit, vegetables, red meat, poultry, fish, eggs, nuts, calcium and iron, zinc, potassium, magnesium, and vitamins A, D, B6, B12, C, E, omega-3, omega-6, protein, and lipid, (*P* = 0.001). While higher tertile of MIND score recorded a lower daily intake of tea and coffee, potatoes, sweets and desserts, sodium, and CHO, (*P* = 0.001).

**TABLE 3:** Dietary intake of macronutrients and components of MIND diet score according to the tertiles (T) of the MIND diet score.

Variables	Tertiles of MIND diet score		
	**T1** **(<6)**	**T2** **(6−7.5)**	**T3** **(7.5−12.5)**
Food groups (g/day)*			
Whole grains	6.00 ± 0.21	9.78 ± 0.24	16.16 ± 0.26**
Refined grains	548.98 ± 2.74	519.43 ± 3.04	478.67 ± 3.26**
Legumes	25.03 ± 0.52	35.11 ± 0.58	46.84 ± 0.62**
Dairy	415.82 ± 6.40	439.95 ± 7.11	492.52 ± 7.62**
Fruits	210.38 ± 3.41	279.93 ± 3.79	358.98 ± 4.07**
Vegetables	335.16 ± 4.00	437.98 ± 4.44	537.79 ± 4.76**
Red meat	26.29 ± 0.56	27.07 ± 0.62	31.19 ± 0.67**
Poultry	36.93 ± 0.64	45.50 ± 0.71	53.38 ± 0.76**
Fish	3.62 ± 0.16	6.18 ± 0.17	10.43 ± 0.19**
Eggs	20.95 ± 0.35	22.60 ± 0.39	21.81 ± 0.42**
Nuts	5.86 ± 0.18	8.71 ± 0.20	12.92 ± 0.21**
Tea and coffee	811.41 ± 8.96	696.35 ± 9.95	637.06 ± 10.67**
Potatoes	51.26 ± 0.69	47.09 ± 0.77	41.82 ± 0.82**
Sweets and desserts	70.0 ± 0.65	59.53 ± 0.72	49.01 ± 0.77**
Butter	4.06 ± 0.088	4.05 ± 0.098	3.35 ± 0.105**
Nutrients*			
Calcium	1232.35 ± 4.95	1237.14 ± 5.50	1248.49 ± 5.89
Iron	17.26 ± 0.06	17.56 ± 0.07	17.67 ± 0.07**
Folate	546.78 ± 2.23	548.78 ± 2.48	545.45 ± 2.66
Sodium	4847.89 ± 25.61	4730.97 ± 28.44	4537.25 ± 30.50**
Zinc	9.13 ± 0.34	9.72 ± 0.38	10.50 ± 0.40**
Potassium	2732.81 ± 13.34	3109.22 ± 14.81	3572.72 ± 15.88**
Magnesium	289.81 ± 0.86	316.32 ± 0.96	348.92 ± 1.03**
Vitamin A	6469.02 ± 86.01	8465.06 ± 95.5	11288.12 ± 102**
Vitamin D	36.63 ± 0.53	45.07 ± 0.59	55.33 ± 0.63**
Vitamin B6	8.96 ± 0.15	11.46 ± 0.17	12.89 ± 0.18**
Vitamin B12	6.00 ± 0.08	7.06 ± 0.09	8.29 ± 0.10
Vitamin C	86.05 ± 0.99	110.23 ± 1.10	140.43 ± 1.18
Vitamin E	6.53 ± 0.05	7.75 ± 0.05	9.00 ± 0.06
Omega 3	0.034 ± 0.0005	0.045 ± 0.0005	0.059 ± 0.0006
Omega 6	4.07 ± 0.05	4.82 ± 0.05	5.23 ± 0.06
**Energy**			
Energy intake (kcal/d)	2372.04 ± 702.1	2576.90 ± 725.92	2750.84 ± 706.62**
Carbohydrate (%E)	62.02 ± 6.32	61.23 ± 6.06	60.55 ± 5.89**
Protein (%E)	12.86 ± 1.95	13.81 ± 1.96	14.70 ± 2.13**
Lipid (%E)	26.57 ± 6.22	26.94 ± 5.91	27.32 ± 5.93**

*The mean ± SD of food parameters is adjusted for daily energy intake.

**The association was statically significant.

Using binary logistic regression analysis, Table 4 shows the relationship between tertials of the MIND diet score and decreased serum HDL, general obesity, and increased serum TG. According to the crude model, those in the third tertile of the MIND diet score were less likely to have increased serum TG and general obesity than those in the first tertile (OR = 0.58; 95% CI 0.38−0.95 and OR = 0.69; 95% CI 0.55−0.91, *P* = 0.001). This result was still significant when confounders were taken into account. Additionally, we found that adhering to the MIND diet increased the probability of having lower HDL-C values (OR = 0.72; 95% CI 0.55−1.15, *P* = 0.001). Even after potential confounders were considered, there was still a significant correlation for lower HDL-C levels (OR = 0.72; 95% CI 0.55−1.15, *P* = 0.001).

**TABLE 4:** The multivariate-adjusted means for HDL and TG according to tertiles (T) of the MIND diet score.

		Odds ratio (95% CI)		
		**T1**	**T2**	**T3**
Increased serum TG	Model I	1	0.76 (0.65, 1.11)	0.58 (0.38, 0.95)*
	Model II	1	0.77 (0.66, 1.12)	0.59 (0.55, 0.70)*
	Model III	1	0.78 (0.67, 1.13)	0.60 (0.43, 1.11)*
Reduced serum HDL	Model I	1	0.94 (0.75, 1.18)	0.72 (0.55, 1.15)*
	Model II	1	0.94 (0.75, 1.18)	0.71 (0.55, 1.15)*
	Model III	1	0.98 (0.81, 1.14)	0.79 (0.47, 1.07)*
General obesity	Model I	1	0.65 (0.57, 0.76)	0.69 (0.55, 0.91)*
	Model II	1	0.66 (0.58, 0.77)	0.78 (0.51, 1.10)*
	Model III	1	0.67 (0.59, 0.78)	0.78 (0.51, 1.10)*

Model I: crude.

Model II: adjusted for age, gender.

Model III: adjusted for age, gender, energy intake, physical activity, SES, smoking, and BMI.

*P*-value < 0.05 obtained by binary logistic regression.

*The association was statically significant.

## Discussion

In this cross-sectional study, we found a significant association between adherence to the MIND diet and general obesity and lipid profile in Kurdish adults. As far as we know, this is the first study that investigates the association between the MIND diet score and general obesity and lipid profile in the adult Kurdish population.

Obesity and being overweight are linked to a higher risk of morbidity and death (19). In recent years, their occurrence has substantially increased (20, 21). Despite knowledge of numerous dietary patterns to avoid chronic illnesses, such eating suggestions seem to be ineffective in preventing obesity. As a result, more effective nutritional methods may be necessary. The MIND diet has recently been proposed as a new healthy eating pattern that is inversely related to the risk of Alzheimer’s disease and cognitive decline (10, 12). As previously stated, the MIND diet is a synthesis of the DASH and MD diets. DASH and MD contain high amounts of dietary fiber than MIND diet because they are rich in fruits, while MIND diet does not contain all fruits included in the DASH and MD diets (22). In addition, although the MIND diet contains beans, it should be noted that owing to nutritional shift, intake of beans in Asian nations’ traditional diets has decreased (23). Therefore, despite the consideration of bean consumption in the MIND diet, the whole bean intake in the diet was not very high. Another difference between the MIND diet and the other two dietary patterns (DASH and MD) is dairy consumption. Earlier studies have shown that greater dairy intake might be associated with a lower risk of obesity (24, 25). This has even been shown in a meta-analysis that weight-loss diets containing high dairy might decrease body weight and fat much more than diets with a low dairy content (26). In the MIND diet, only cheese consumption was included while in the DASH and MD diets, consumption of all dairy products is encouraged.

One of the important findings of this study was the inverse significant association between the MIND diet and the odds of low serum HDL-C and elevated serum TG, but no significant association was observed between TC and LDL-C. Mohammadpour et al. (27) showed that the MIND diet score is inversely associated with probabilities of lower HDL in a cross-sectional study of 836 Iranian individuals. There is no other study examining the linkage between the MIND diet and lipid profile but Azadbakht et al. (28) showed that an 8-week randomized study (*n* = 31) on T2D patients might enhance HDL cholesterol by following the DASH diet. In contrast, in the study of Obarzanek et al. (29) the DASH diet showed reduced HDL-C, which is explicable by lower consumption of total dietary fat.

Following adherence to the MIND diet, we also saw a significant decline in general obesity in our study. In this area, the evidence is contradictory. According to their findings, Mohammadpour et al. (27) suggested a beneficial effect of MD on general obesity. In contrast, Aminianfar et al. (22) found that according to their study, there are no causal links between following the MIND diet and the risk of both central and general obesity in either men or women. However, the sample size was modest and olive oil was not factored into their evaluation. Esposito and others (16) reported that energy restriction accelerated MD-induced weight loss and that MD had a positive effect on body weight independent of energy consumption. Another meta-analysis revealed that following the DASH diet effectively decreased body weight, BMI, and waist circumference (approximately 1.42 kg in 8–24 weeks), especially when paired with a low–energy diet (30).

Calcium’s positive impact on fat accumulation prevention may be attributed to the expression of the uncoupled protein (UCP2) in white adipose tissue, which boosts thermogenesis and reduces waist circumference (31). Furthermore, casein and conjugated linolenic acid could help reduce the accumulation of fat (32).

Fruits are an excellent source of phytochemicals, fiber, and antioxidants that may help with weight control (32), inhibit the development of reactive oxygen species and systemic oxidative damage (33, 34). Berries are an excellent source of flavonoids, tannins, phenolic acids, and lignans, among other polyphenols (35). Thirty middle-aged men with abdominal obesity received three different doses (125, 250, and 500 ml/day) of cranberry juice for 4 weeks from Ruel et al. (36). After drinking 250 ml and/or 500 ml, they discovered that waist circumference, BMI, weight, total/HDL cholesterol ratio, HDL cholesterol, apolipoprotein B, and plasma antioxidant capacity were all significantly lower.

The major source of dietary fat and phenolic compounds on the MIND diet may have been olive oil, which also has other health benefits. George et al. (37) found that oxidative stress markers and HDL cholesterol may both be improved by high polyphenol olive oil. Tsartsou et al. (38) indicated that the primary impact of high polyphenol content olive oil is to raise HDL cholesterol levels in the blood. The decrease in overall obesity shown in this research may be due to a few MIND diet components. For example, consuming foods in the third tertile of the MIND diet score has minimal calories (39). This diet also contains plant-based meals with low glycemic loads, high fiber content, and water content (40). Consequently, it promotes weight reduction (41, 42).

The analysis was performed on a large sample size, and statistical controlling for potential confounders is among the strengths of the present study. Also, the major limitation of the present study is its cross-sectional design, which prohibits inferring causal relationships between the MIND diet and general obesity and lipid profile.

## Conclusion

Significant associations were found between adherence to the MIND diet and odds of general obesity and lipid profile in this cross-sectional study among Kurdish adults. This finding suggests that despite the usefulness of the MIND diet for having a healthy brain it might be able to predict other chronic conditions like obesity. Given the limitations we had in the study, investigations are needed to further examine the association between this dietary pattern and the risk of obesity.

## Data availability statement

The raw data supporting the conclusions of this article will be made available by the authors, without undue reservation.

## Ethics statement

The studies involving human participants were reviewed and approved by the Ethics Committee of Sulaimani Polytechnic University, Kalar Technical College approved the study (No: KTC: 0629092022). The procedure for obtaining informed consent was approved by the Ethics Committee of Sulaimani Polytechnic University, Kalar Technical College. All methods were carried out by relevant guidelines and regulations. This study was conducted by the Declaration of Helsinki. The patients/participants provided their written informed consent to participate in this study. Written informed consent was obtained from the individual(s) for the publication of any potentially identifiable images or data included in this article.

## Author contributions

HF and NK designed the study. SM and HF collected and analyzed the data. All authors prepared the draft of the manuscript, contributed to the article, and approved the submitted version.
